# Cardiac evaluation in adults with dengue virus infection by serial echocardiography

**DOI:** 10.1186/s12879-021-06639-x

**Published:** 2021-09-10

**Authors:** Chayasin Mansanguan, Borimas Hanboonkunupakarn, Sant Muangnoicharoen, Arun Huntrup, Akkapon Poolcharoen, Suyanee Mansanguan, Watcharapong Piyaphanee, Weerapong Phumratanaprapin

**Affiliations:** 1 Department of Clinical Tropical Medicine, Faculty of Tropical Medicine, Mahidol University, Bangkok, Thailand; 2 Hospital for Tropical Diseases, Faculty of Tropical Medicine, Mahidol University, Bangkok, Thailand; 3 Bhumibol Adulyadej Hospital, Bangkok, Thailand

**Keywords:** Dengue infection, Cardiac involvement, Haemodynamic, Myocarditis, Serial echocardiography

## Abstract

**Background:**

Dengue virus infection (DVI) is a major health problem in many parts of the world. Its manifestations range from asymptomatic infections to severe disease. Although cardiac involvement has been reported in DVI, its incidence has not yet been well established.

**Methods:**

From July 2016 to January 2018, patients hospitalized at the Hospital for Tropical Diseases, Faculty of Tropical Medicine, Mahidol University, Thailand, with dengue virus infection confirmed by positive NS1 or positive dengue immunoglobulin M findings, participated in the study. We characterized the incidence and change in cardiac function by serial echocardiography and levels of troponin-T and creatine kinase-myocardial band (CK-MB) on the day of admission, the day of defervescence, the first day of hypotension (if any), and at 2 week follow-up.

**Results:**

Of the 81 patients evaluated, 6 (7.41%) exhibited elevated biomarker levels. There was no difference in clinical presentation amongst dengue fever, dengue haemorrhagic fever (DHF) and dengue shock syndrome (DSS), except for the amount of bleeding. Cardiac involvement was found in 22.2% of patients: 3 (3.70%) had left ventricular systolic dysfunction, 3 (3.70%) had transient diastolic dysfunction, 6 (7.41%) had increased levels of at least one cardiac biomarker (troponin-T or CK-MB), and 6 (7.41%) had small pericardial effusion. Myocarditis was suspected in only two patients (with DHF); thus, myocarditis was uncommon in patients with dengue virus infection. Three patients developed DSS during admission and were transferred to the intensive care unit.

**Conclusion:**

Cardiac involvement in adults with dengue infection was common, ranging from elevated cardiac biomarker to myocarditis. Abnormalities in cardiac function had resolved spontaneously by the day of follow-up, without specific treatment. We found that DHF was a significant risk factor for cardiac involvement. Echocardiography is the investigation of choice for evaluating the haemodynamic status of patients with DVI, especially in severe dengue.

**Supplementary Information:**

The online version contains supplementary material available at 10.1186/s12879-021-06639-x.

## Background

Dengue virus infection (DVI) is a major health problem in more than 100 countries in tropical and subtropical regions. Approximately 96 million people develop DVI annually [1]. DVI has a broad clinical spectrum, according to classification by the World Health Organisation (WHO, 2009), which includes asymptomatic to dengue fever, dengue haemorrhagic fever (DHF), and dengue shock syndrome (DSS) [2]. Cardiac complications are amongst the important consequences of DVI.

The manifestations and frequency of cardiac involvement in dengue are difficult to describe and define. The actual incidence of, and details about, cardiac involvement in Thailand are neither well-described nor well-defined. Among studies, the incidence of cardiac involvement in DVI varies from 15 to 40% [3–6]. Various forms of cardiac involvement in DVI include transient atrioventricular block, relative bradycardia and myocarditis; patients may also develop acute pulmonary edema or cardiogenic shock [5–7]. Although severe cardiac complications, such as myocarditis, have been reported in the literature, few sectional or cohort study studies have been during DVI [8–10]. In China, the prevalence of myocarditis in the worst dengue outbreak was 11.28%, and evidence of myocarditis increased with disease severity [11]. In Thailand, Wiwanitkit reviewed previous reports of dengue myocarditis and found only 2 patient reports and 4 autopsy cases from over 6,000 Thai DHF cases. The author suggested that the small number of reports could be due to its rare manifestation or underdiagnosis [12]. Dengue epidemics vary in severity, so previously reported frequencies may not represent an accurate assessment. Cardiac involvement in DVI must be better understood. In this study, we aimed to characterise, describe and evaluate the dynamics of cardiac function using serial echocardiography in patients with different clinical manifestations of DVI over a period of several years. We also aimed to evaluate the risk factors for cardiac involvement in patients with DVI.

## Methods

### Ethical considerations

The study design was approved by the Ethics Committee of the Faculty of Tropical Medicine, Mahidol University (Certificate No. MUTM 2016-005-02). Written informed consent was obtained from patients before enrolment into the study.

### Study design

This prospective study focused on adult patients with dengue admitted to the Hospital for Tropical Diseases, Faculty of Tropical Medicine, Mahidol University, Bangkok, Thailand, between July 2016 and January 2018. The study’s inclusion criteria were: (1) adult, at least 18 years old; (2) having DVI confirmed by either a positive result of dengue non-structural protein 1 (NS1) testing or the presence of dengue immunoglobulin M (IgM) antibodies in acute-phase sera by enzyme-linked immunosorbent assay (ELISA). Patients who had had myocardial infarction within the previous month or history of cardiomyopathy were excluded from the study. Laboratory investigations, including complete blood count, blood chemistry, troponin-T, CK-MB, ECG and 2D echocardiography, were performed on the day of admission, day of defervescence, the first day of hypotension (if any), and at 2 weeks’ follow-up. During admission, patient’s haematocrit and platelet count were measured once a day, at minimum. The frequencies of measurements increased during the critical phase to every 4–8 h. or according to the physician’s judgement. Patients’ data, including demographic data, clinical presentation, and laboratory findings, were recorded in a pre-defined case-record form.

### Definition of cardiac involvement

In this study, cardiac involvement was defined as one or more of the following:

1. Left ventricular systolic dysfunction: defined as systolic dysfunction referring to impaired ventricular contraction (left ventricular ejection fraction; LVEF less than 50%)
2. Transient diastolic dysfunction: Diastolic dysfunction is an impaired left ventricular relaxation with increased stiffness of the LV and elevated filling pressure. Diastolic dysfunction is divided into 4 grades (I, II, III, and IV) according to an update from the American Society of Echocardiography [13].
3. Myocarditis compatible with the ESC myocarditis criteria [14]: defined by positive specific cardiac biomarker (troponin-T and/or CK-MB) associated with depressed LVEF less than 50% by echocardiography.
4. Pericarditis or pericardial effusion: Pericarditis characterized by chest pain and abnormal ECG finding
5. Elevated levels of at least one cardiac biomarker (troponin-T or creatine kinase–myocardial band [CK-MB])

### General definitions

An adult is defined as a person aged 18 years or older. Obesity [15] is defined as a BMI > 27.5 kg/m^2^ and overweight is defined as a BMI > 23 kg/m^2^, as adjusted for Asian population parameters. Severe transaminitis is defined as elevated aspartate aminotransferase (AST) or alanine aminotransferase (ALT), or both > 10 × the upper normal limit.

### Case definition of severe dengue

WHO (2009) case definitions were used for severe DVI [16]. Severe dengue was classified as having: (1) severe plasma leakage that necessitated fluid resuscitation from shock, (2) severe clinical bleeding, defined as spontaneous bleeding from the mucosal area that needed blood transfusion, or bleeding in the vital organs, (3) evidence of organ involvement, including heart failure or myocarditis, AST or ALT levels > 1000 IU/L, and impaired consciousness.

### Definition of severity of dengue fever

Patients were confirmed with DVI were classified on the basis of WHO 1997 dengue case definition [2], into dengue fever (DF), dengue haemorrhagic fever (DHF), and dengue shock syndrome (DSS), based on clinical and laboratory criteria. Four cardinal features of DHF, as defined by the WHO, are as follows: (1) fever or history of fever lasting 2–7 days, occasionally biphasic, (2) haemorrhagic tendencies, evidenced by at least one of the following: positive tourniquet test; petechiae, ecchymoses or purpura; bleeding from the mucosa, gastrointestinal tract, injection sites or other locations; haematemesis or melena, (3) thrombocytopenia (100,000 cells per mm^3^ or less), (4) evidence of plasma leakage owing to increased vascular permeability shown by: an increase in haematocrit > 20% above average for age, sex and population; a decrease in haematocrit after intervention > 20% of baseline; signs of plasma leakage, such as pleural effusion, ascites or hypoproteinemia.

Dengue shock syndrome (DSS) as defined by all four criteria for DHF must be met, in addition to evidence of circulatory failure manifested by: rapid and weak pulse and narrow pulse pressure (< 20 mmHg or 2.7 kPa) manifested by hypotension for age, and cold, clammy skin and restlessness or lethargy.

### Cardiac enzymes

Troponin-T hs assay and CK-MB isoenzyme levels were determined for all dengue patients during hospitalization on day of admission, day of defervescence, and 2 weeks after discharge (early convalescence). Troponin-T was measured using with the Elecsys troponin-T hs assay (Roche Diagnostics, Mannheim, Germany) and serum levels > 40 pg/mL were considered elevated. CK-MB was also measured by enzyme-linked fluorescent assay (MyBioSource, San Diego, CA, USA) and serum CK-MB levels > 3.77 ng/mL were considered elevated. All tests were performed in batches after study completion and by clinicians without knowledge of the clinical diagnosis.

### Chest radiography

Chest radiography was performed by an experienced technician on the day of defervescence and was read by a radiologist with 30 years’ experience.

### Echocardiography protocol

Using the Vivid E9 ultrasound platform (GE Healthcare, Chicago, IL, USA), a single cardiologist with almost 10 years’ experience performed echocardiography from the time of patient enrolment into the study to the 2 week follow-up visit. The cardiologist was blinded to the laboratory results.

All images were recorded and analyzed according to a predefined method with the same software analysis system (GE Healthcare). Systolic and diastolic blood pressure and the results of electrocardiography (ECG) were recorded during the examination.

All echocardiographic images were recorded and reviewed by a single operator. Routine two-dimensional echocardiograms and colour-flow Doppler images were obtained in the standard parasternal long axis view, subcostal view, apical two-chamber views, and apical four-chamber views. The left ventricular walls and dimensions were measured in accordance with the guidelines of the American Society of Cardiology. Transmitral pulsed-wave Doppler velocities (peak E- and A-wave velocities) were measured in the apical four-chamber view with the sample volume positioned at the mitral valve. Tissue Doppler imaging of the left ventricle was performed with pulsed-wave Doppler assessment of the medial and lateral mitral valve annulus, peak tissue medial and lateral.

The diameter of the inferior vena cava (IVC) was measured for use in determining the CVP level in the subcostal view. In the subcostal view, IVC was visualized as it entered the right atrium. The M-mode was used to create a time-motion image of the IVC diameter (IVCd) and collapsibility index (IVCc). The minimum IVC diameter (IVCdmin) was measured at the end-inspiratory phase. The maximum IVC diameter (IVCdmax) was measured at the end-expiratory phase over one respiratory cycle. Pericardial effusion and other anatomical and functional findings were recorded when present. Echocardiographic studies were performed on the day of admission, day of defervescence, the first day of hypotension (if any), and at 2 week follow-up. Cardiologists were available every day.

### Sample-size calculation

To calculate the necessary sample size, we used the estimated prevalence described in a previous study [17], where the incidence of cardiac involvement was 37% among adults with dengue infection. Based on this information, a minimum sample size of 63 was sufficient for this study to determine cardiac involvement, with an error of 12% at 95% confidence interval. Cardiac biomarkers and echocardiography followed up at 2 weeks. We expected 10% of patients could refuse to participate or drop out before the study’s end. Thus, a sample size of at least 70 patients with dengue was required for this study. Due to the dengue season we were able to collect 81 cases before study end.

### Statistical analysis

All data were analyzed using SPSS version 18.0 (IBM, Armonk, NY, USA). Qualitative variables were calculated as frequencies and percentages. In the descriptive part of the analysis, the categorical variables were demonstrated as frequency and percentage. Chi-square test or Fisher’s exact test with corresponding p-value were used, as appropriate, to find associations among categorical data. Continuous data were expressed as median with inter-quartile range (IQR) and mean with standard deviation, depending on their data distribution. Student’s t-test, pair t-test, and Mann–Whitney test, were used to determine the difference in mean or median among two groups in continuous data. A multivariate logistic regression model was used to determine the association of independent factors of cardiac involvement (adjusted odds ratio [OR] with 95% confidence interval). All tests of significance were two-sided tests, with “*p*” value < 0.05 indicating statistical significance.

## Results

**Fig. 1:**
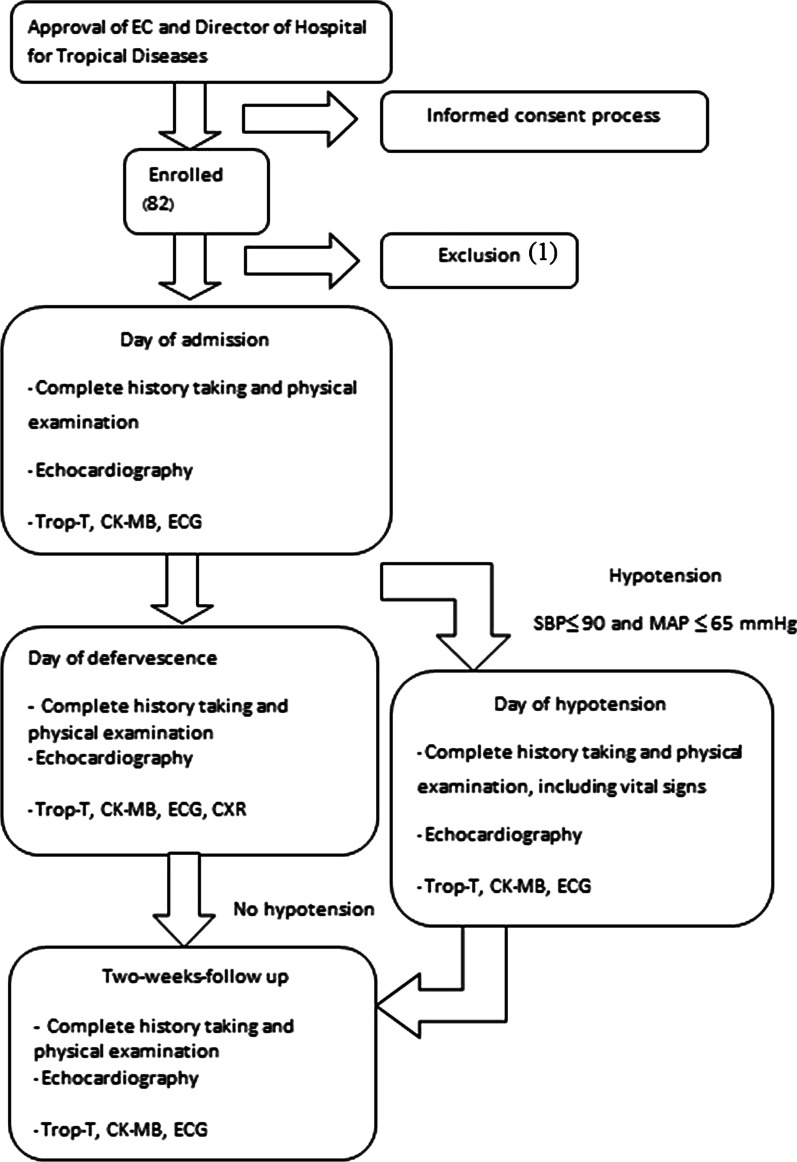
Study flow chart. *Trop-T* Troponin-T; *CK-MB* creatine kinase–myocardial band; *ECG* Electrocardiography

A total of 81 patients hospitalized with DVI between July 2016 and January 2018 were included in the study, as shown in Fig. 1

### Characteristics of the patients

The mean age of the patients was 33 years (SD 13 years; range 20–46 years). All patients presented with the typical clinical manifestations of DVI; symptoms had begun a mean of 4 days earlier. 12 patients had underlying medical conditions–diabetes mellitus, hypertension, dyslipidaemia, and others. The detailed of current medication in patients with underlying medical condition is in theAdditional file 1. Of the 81 patients, 39 (48.15%) were classified with dengue fever, 39 (48.15%) as grade I or II DHF, and 3 (3.70%) as grade III or IV DHF. DVI was confirmed by the detection of NS1 antigen in 71 patients (87.65%) and by the presence of specific IgM antibody in 37 (45.68%). The clinical and laboratory characteristics of all 81 patients are shown in Table 1.

**Table 1:** Demographic, clinical and laboratory characteristics of 81 patients with dengue virus infection at admission

Characteristic	Total (*N* = 81)	DF (*n* = 39)	DHF (*n* = 42)	*p*-value
Sex				0.418^a^
Female (n, %)	44 (54.32%)	23 (58.97%)	21 (50.00%)	
Male (n, %)	37 (45.68%)	16 (41.03%)	21 (50.00%)	
Age (years), mean ± SD	32.95 ± 13.56	33.79 ± 14.46	32.17 ± 12.8	0.592^b^
Body mass index (BMI), (kg/m^2^), mean ± SD	22.77 ± 4.93	21.99 ± 4.71	23.5 ± 5.07	0.170^b^
Nutritional status per classification for Asian				0.721^a^
Underweight (n, %)	14 (17.3)	8 (20.5)	6 (14.3)	
Normal weight (n, %)	35 (43.2)	18 (46.2)	17 (40.5)	
Overweight (n, %)	17 (21.0)	7 (17.9)	10 (23.8)	
Obesity (n, %)	15 (18.5)	6 (15.4)	9 (21.4)	
Underlying diseases (n, %)	12 (14.8%)	5 (12.8%)	7 (16.7%)	0.758^c^
Diabetes mellitus (n, %)	2 (2.5%)	2 (5.1%)	0 (0%)	0.229^c^
Hypertension (n, %)	3 (3.7%)	3 (7.7%)	0 (0%)	0.229^c^
Dyslipidaemia (n, %)	3 (3.7%)	2 (5.1%)	1 (2.4%)	0.606^c^
Mucosal bleeding (n, %)	35 (43.21%)	12 (30.8%)	23 (54.76%)	0.029^a^
Fever duration (days), mean ± SD	3.83 ± 1.17	3.95 ± 1.05	3.71 ± 1.27	0.371^b^
Diagnostic test for dengue infection				
NS1 antigen positive (n, %)	70 (95.9%)	32 (97.0%)	38 (95.0%)	0.999^c^
NS1 antigen negative (n, %)	3 (4.1%)	1 (3.0%)	2 (5.0%)	
IgM antibody positive (n, %)	23 (35.4%)	11 (33.3%)	12 (37.5%)	0.798^c^
IgM antibody negative (n, %)	42 (64.6%)	22 (66.7%)	20 (62.5%)	
IgG antibody positive (n, %)	37 (56.1%)	18 (54.5%)	19 (57.6%)	0.999^c^
IgG antibody negative (n, %)	29 (43.9%)	15 (45.5%)	14 (42.4%)	
General laboratory investigation				
WBC, median (IQR), × 10^3^/μL	4.5(3.5,5.7)	4.5(3.2,5.8)	4.7(3.6,5.6)	0.833^d^
Haemoglobin median (IQR), g/dL	13.4 (12.5, 14.9)	13.3 (12.5, 14.7)	13.9 (12.4, 14.9)	0.620^d^
Haematocrit, median (IQR), %	40.2 (37.0, 43.1)	39.5 (37.1, 42.9)	41.1 (36.3, 43.5)	0.702^d^
Platelet counts, median (IQR), × 10^3^/μL	53.0(30.0,81.5)	59.0(33.0,90.0)	40.5(25.8,73.3)	0.040^d^
Creatinine, median (IQR), mg/dl	0.73 (0.61, 0.84)	0.75 (0.61, 0.89)	0.73 (0.60, 0.82)	0.280^d^
Maximum AST, median (IQR), IU/L	112.5 (47.7,282.0)	107.5(46.3, 190.5)	132.0(56.5, 407.0)	0.236^d^
Maximum ALT, median (IQR), IU/L	83.5 (29.8, 201.3)	78 (31, 129)	88.5 (29.3, 290.3)	0.341^d^
Cardiac involvement characteristic and investigation				
Left ventricular systolic functions, mean ± SD, %	69.04 ± 7.32	68.9 ± 5.77	69.17 ± 8.58	0.868^c^
Left ventricular diastolic functions				
MV-E, mean ± SD, cm/s	0.86 ± 0.2	0.88 ± 0.2	0.84 ± 0.21	0.344^c^
MV-A, mean ± SD, cm/s	0.63 ± 0.17	0.64 ± 0.18	0.61 ± 0.17	0.481^c^
MV-E/A, mean ± SD	1.49 ± 0.54	1.5 ± 0.54	1.48 ± 0.54	0.867^c^
Troponin-T elevation (n, %)	3 (3.7%)	0 (0%)	3 (7.10%)	0.242^a^
CK-MB elevation (n, %)	5 (6.2%)	0 (0%)	5 (11.9%)	0.056^d^
Cardiac involvement (n, %)	18 (22.2%)	1 (2.6%)	17 (40.5%)	< 0.001^c^
Pericardial effusion (n, %)	6 (33.3%)	0 (0%)	6 (35.3%)	
Transient systolic dysfunction (n, %)	3 (16.7%)	1 (100%)	2 (11.8%)	
Transient diastolic dysfunction (n, %)	3 (16.7%)	0 (0%)	3 (17.6%)	
Elevated biomarker (n, %)	4 (22.2%)	0 (0%)	4 (23.5%)	
Elevated biomarker with myocarditis (n, %)	2 (11.1%)	0 (0%)	2 (11.8%)	

^a^Pearson Chi-square test; ^b^independent t test; ^c^Fisher’s Exact test; ^d^Mann-Whitney U test; *IQR* interquartile range; *WBC* white blood cell count; *AST* aspartate aminotransferase; *ALT* alanine aminotransferase; *MV-E* MV flow E-wave velocity; *MV-A* MV flow A-wave velocity; *MV-E/A* the E(early) to A(late) ratio ventricular velocities; *CK-MB* creatine kinase–myocardial ban

### Comparison between patients with and without elevated cardiac biomarkers

In the majority of patients (75 [92.5%]), cardiac biomarker levels were not elevated. Patients whose biomarker levels were elevated had significantly more cardiac involvement in DVI (*p* < 0.001). The characteristics of the patients with increased levels of troponin-T, CK-MB or both, and of patients with normal levels, are listed in Table 2.

**Table 2:** Comparison between patients with and without elevation in biomarker levels

Characteristics	With biomarker elevation (*n* = 6)	Without biomarker elevation (*n* = 75)	p-value
Age, (years), mean ± SD	28.7 ± 6.2	33.3 ± 14.0	0.156^a^
Male sex (n,%)	5 (83.3%)	32 (42.7%)	0.088^c^
Dengue classification			0.026^c^
Dengue fever (n,%)	0 (0%)	39 (52.0%)	
Dengue haemorrhagic fever (n,%)	6 (100%)	36 (48.0%)	
Grade I or II (n, %)	4 (66.7%)	35 (46.7%)	
Grade III or IV (n, %)	2 (33.3%)	1 (1.3%)	
Underlying diseases (n, %)	2 (33.3%)	10 (13.3%)	0.215^c^
Diabetes mellitus (n, %)	0 (0%)	2 (2.7%)	0.999^c^
Hypertension (n, %)	0 (0%)	2 (2.7%)	0.999^c^
Dyslipidaemia (n, %)	1 (16.7%)	2 (2.7%)	0.209^c^
Fever duration (days), mean ± SD	3.5 ± 1.38	3.85 ± 1.16	0.480^a^
WBC, median (IQR), × 10^3^ /μL	5.3 (3.9,9.5)	4.5(3.3,5.7)	0.283^d^
Haemoglobin, median (IQR), g/dL	14.6 (11.2, 15.2)	13.3 (12.5, 14.8)	0.780^d^
Haematocrit, median (IQR), %	42.2 (31.6, 42.9)	39.7 (37.1, 43.1)	0.964^d^
Platelet counts, median (IQR), × 10^3^/μL	60.0(27.3,83.8)	53.0(30.0,83.3)	0.857^d^
Creatinine, median (IQR), mg/dl	0.81 (0.67, 1.50)	0.72 (0.61, 0.83)	0.199^d^
Troponin-T elevation (n,%)	3 (50.0%)	0 (0%)	< 0.001^c^
CK-MB elevation (n,%)	5 (83.3%)	0 (0%)	< 0.001^c^
Patients with cardiac involvement (n,%)	6 (100%)	12 (16%)	< 0.001^c^

^a^Pearson Chi-square test; ^b^independent t test; ^c^Fisher’s Exact test; ^d^Mann-Whitney *U* test;

*IQR* interquartile range, *WBC* white blood cell count

### Evaluation of patients with cardiac involvement

**Fig. 2:**
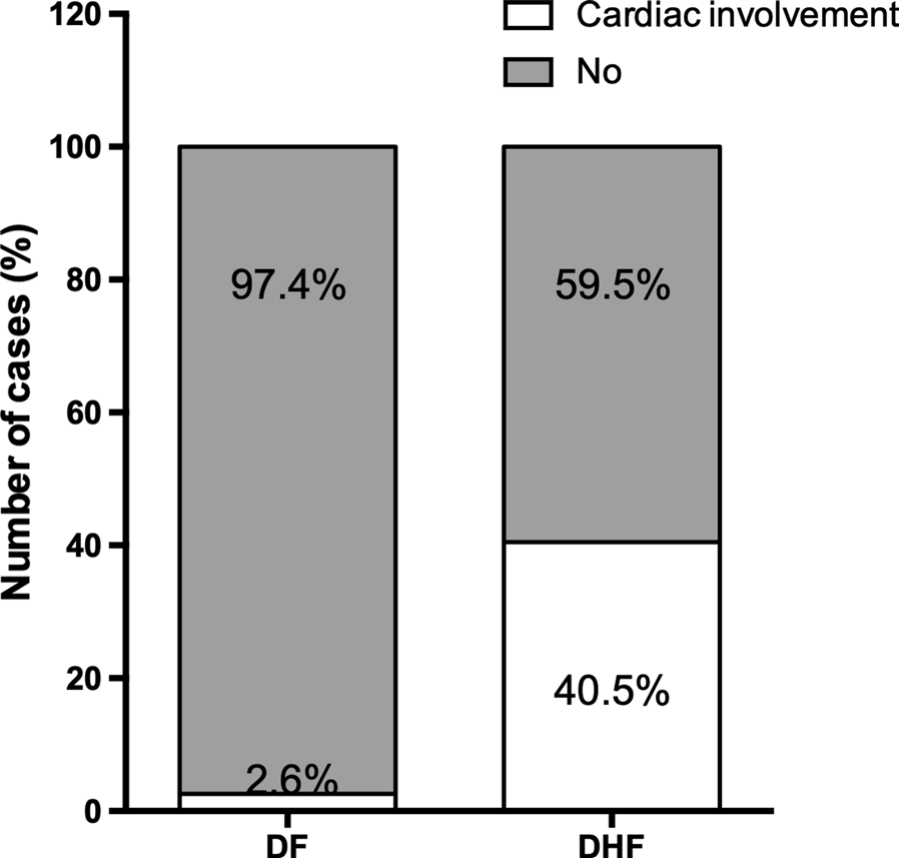
Percentages of DF/DHF patients with and without cardiac involvement. *DF* dengue fever, *DHF* dengue haemorrhagic fever

Of the 81 patients with DVI, 18 (22.2%) had cardiac involvement (Fig. 2): 3 (3.70%) had left ventricular systolic dysfunction, 3 (3.70%) had transient diastolic dysfunction, 6 (7.41%) had increased levels of at least one cardiac biomarker (troponin-T or CK-MB), and 6 (7.41%) had small pericardial effusion. Of the 42 patients with DHF, 17 (40.5%) had cardiac involvement. We found that cardiac involvement was more common in DHF than in dengue fever.

**Fig. 3:**
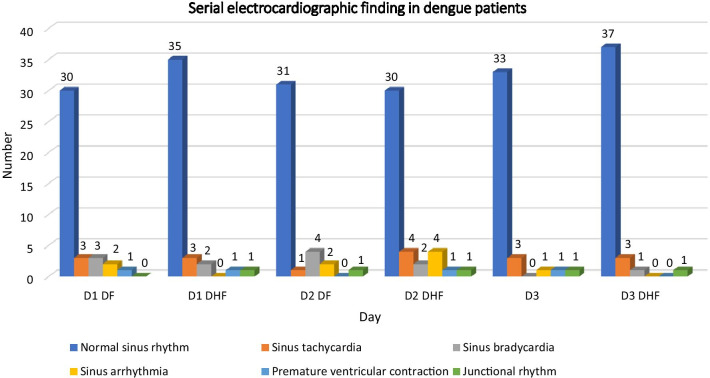
Serial ECG finding of DF/DHF patients on day of admission (day1), on day of defervescence (day 2: critical period) and on day of 2 week follow-up (day 3)

On admission, ECG revealed that, of the 81 patients, 78 (96.30%) had normal sinus rhythm. Of the three patients with abnormal rhythm, two (2.47% of the total) had junctional rhythm and one (1.23%) had premature ventricular contractions (Table 3). Serial ECG findings are shown in Fig. 3. At day of defervescence, ECG findings revealed normal ECG finding in 61(75.3%) of patients while abnormal ECG finding was noted in 20 (24.7%). At 2 weeks’ follow-up, ECG findings revealed normal ECG finding in 70 (86.4%) of patients, while abnormal ECG findings were noted in 11 (13.6%).

**Table 3:** Electrocardiographic finding at the time of admission

Rhythm	Number (*N* = 81)
Normal sinus rhythm	65 (80.25%)
Sinus tachycardia	6 (7.41%)
Sinus bradycardia	5 (6.17%)
Sinus arrhythmia	2 (2.47%)
Premature ventricular contraction	2 (2.47%)
Junctional rhythm	1 (1.23%)

### Evaluation of patients with biomarker elevation

The cardiac manifestations, serial cardiac biomarker characteristics of the 6 patients (7.41%) with elevated cardiac-biomarker levels, are shown in Table 4. Four (4.94%) patients presented with clinical manifestations of cardiac involvement: myocarditis in two patients, acute heart failure with shock in one patient, and shock in two patients. Two patients had suspected myocarditis. These patients were young (33 and 37 years) and presented without underlying disease. Myocarditis was confirmed by depressed LVEF in echocardiography, and elevated cardiac enzymes (troponin-T and CK-MB). Dengue myocarditis is not common, and cardiac enzyme(s) may not be elevated among some dengue patients with cardiac involvement but without myocarditis.

**Table 4:** Characteristic of patients with elevated level of biomarkers (Troponin-T or CK-MB)

Demographic				Clinical presentation										Echocardiography				
**No**	**Age (y)**	**Sex**	Symptom duration (days)	Classification	Cardiac manifestation	CK-MB level (ng/mL)				Troponin-T (pg/mL)				LVEF				Pericar-dialeffusion
						1	2	3	4	1	2	3	4	1	2	3	4	
1	< 20	Male	3	DHF	None	323	2.88	–	< 1.0	< 50	< 50	–	< 50	64	73	–	62	No
2	26–30	Male	4	DHF	Shock	15.8	4.52	2.89	2.58	< 50	< 50	< 50	< 50	62	65	63	64	No
3	26–30	Male	6	DHF	None	1.01	1.0	–	1.4	< 50	50–100	–	< 50	62	71	–	78	No
4	26–30	Male	2	DHF/DSS	Heart failure, shock	1.85	19	19	3.8	< 50	< 50	< 50	< 50	61	80	80	74	Yes
5	36–40	Male	3	DHF	Myocarditis	2.33	18.2	–	14.9	< 50	1344	–	962	69	50	–	69	No
6	31–35	Female	3	DSS	Myocarditis, shock	7.55	9.78	5.73	1.35	392	1210	1456	139	57	45	50	76	Yes

*CK-MB* creatine kinase–myocardial band, *DHF* dengue haemorrhagic fever, *DSS* dengue shock syndrome, *LVEF* left ventricular ejection fraction. 1, the day of admission; 2, the day of defervescence; 3, the first day of hypotension; 4, 2 week follow up day

### Independent factors associated with cardiac involvement

Univariate analysis revealed the following independent variables: age, male sex, overweight, obesity, underlying disease, DHF, severe transaminitis, and severe dengue. Categorical variables were placed into the model with entry at 0.05 and removal at 0.10 and were scored with ‘no’ as the reference category. In this way, two variables were eliminated: severe transaminitis and obesity. Eventually, DHF was identified as the only risk factor for the development of cardiac involvement in DVI (p < 0.001; Table 5).

**Table 5:** Independent associated factors for cardiac involvement, according to univariate and multivariate analyses

	Crude OR (95% CI)	*p*-value	Adjusted OR (95% CI)	*p*-value
Age	1 (0.96–1.04)	0.872		
Male sex	3.04 (1.01–9.15)	0.048*	2.97 (0.85–10.42)	0.089
Overweight	1.3 (0.45–3.75)	0.627		
Obesity	1.51 (0.41–5.56)	0.532		
Underlying diseases	1.96 (0.52–7.47)	0.322		
DHF	25.84 (3.23–206.63)	0.002*	22.99 (2.78–190.26)	0.004*
Severe dengue	5.67 (1.34–24.07)	0.019*	2.6 (0.52–12.84)	0.242
Severe transaminitis	0.27 (0.07–1.04)	0.056		

*Statistically significant by two-tailed *p*-value (<0.005)

*CI* confidence interval, *DHF* dengue haemorrhagic fever, *OR* odds ratio

All patients underwent a follow-up examination during early convalescence (< 2 weeks post discharge). Trends towards improvement from day of defervescence to convalescent phase were found for LVEF, stroke volume, and cardiac index, in cases of dengue fever and dengue haemorrhagic fever (Table 6).

**Table 6:** Comparison between cardiac function on day of admission (day 1), day of defervescence (day 2: critical period) and on day of 2 week follow-up (day 3)

Cardiac function	Dengue fever					Dengue haemorrhagic fever				
	Day 1	Day 2	Day 3	*p*-value D1 VS D2	*p*-value D2 VS D3	Day 1	Day 2	Day 3	*p*-value D1 VS D2	*p*-value D2 VS D3
Heart rate, mean ± SD	81.14 ± 15.28	70.09 ± 10.25	73.42 ± 11.71	< 0.001*	0.072	79.32 ± 11.27	70.43 ± 13.85	74.65 ± 10.01	< 0.001*	0.066
MV flow E-wave velocity, mean ± SD	0.88 ± 0.2	0.86 ± 0.17	0.91 ± 0.17	0.066	0.064	0.83 ± 0.21	0.83 ± 0.2	0.94 ± 0.17	0.797	0.001*
MV flow deceleration time, mean ± SD	179.27 ± 35.81	184.2 ± 36.85	175.71 ± 36.33	0.880	0.514	169.43 ± 45.64	194.06 ± 48.06	175.73 ± 38.84	0.023*	0.392
MV flow A-wave velocity, mean ± SD	0.64 ± 0.18	0.58 ± 0.22	0.63 ± 0.18	0.002*	0.022*	0.61 ± 0.17	1.59 ± 6.56	0.71 ± 0.2	0.342	0.379
MV annulus E-wave velocity, mean ± SD	1.5 ± 0.54	2.25 ± 3.4	1.79 ± 1.21	0.246	0.500	1.48 ± 0.54	1.63 ± 0.58	1.42 ± 0.46	0.065	0.053
E/A ratio, mean ± SD	1.51 ± 0.54	1.68 ± 0.63	1.59 ± 0.51	0.038	0.428	1.47 ± 0.54	1.63 ± 0.58	1.42 ± 0.46	0.051	0.053
e´, mean ± SD	0.12 ± 0.04	0.13 ± 0.07	0.13 ± 0.08	0.385	0.890	0.11 ± 0.03	0.1 ± 0.03	0.12 ± 0.03	0.587	0.051*
E/e´, mean ± SD	8.01 ± 2.09	8.09 ± 2.49	8.42 ± 2.42	0.797	0.295	8.34 ± 2.56	8.38 ± 2.02	8.35 ± 2.35	0.840	0.938
a´, mean ± SD	0.09 ± 0.02	0.07 ± 0.02	0.09 ± 0.02	0.009*	0.001*	0.08 ± 0.03	0.08 ± 0.03	0.08 ± 0.03	0.345	0.202
LVEF, mean ± SD	68.9 ± 5.77	69.4 ± 9.28	72.12 ± 7.63	0.822	0.022*	69.17 ± 8.58	68.68 ± 7.08	71.35 ± 6.82	0.835	0.086
LVOT mean velocity,mean ± SD	0.59 ± 0.13	0.55 ± 0.13	0.57 ± 0.12	0.081	0.063	0.57 ± 0.12	0.52 ± 0.13	0.58 ± 0.13	0.087	0.024*
Atrioventricular mean velocity, mean ± SD	0.88 ± 0.18	0.78 ± 0.14	0.83 ± 0.18	0.005*	0.018*	0.83 ± 0.14	0.82 ± 0.28	0.83 ± 0.18	0.187	0.619
Left atrial diameter, mean ± SD	3.44 ± 0.48	3.43 ± 0.43	3.47 ± 0.4	0.664	0.402	3.34 ± 0.54	3.53 ± 0.47	3.56 ± 0.48	0.026*	0.894
Cardiac index, mean ± SD	3.54 ± 1.18	2.98 ± 0.89	3.27 ± 0.95	0.003*	0.039*	3.26 ± 0.83	2.9 ± 0.84	3.29 ± 1.03	0.064	0.030*
Stroke volume, mean ± SD	69.47 ± 16.88	69.22 ± 18.14	71.56 ± 22.01	0.596	0.093	67.9 ± 16.22	69.79 ± 20.97	74.11 ± 19.03	0.429	0.233
IVC collapse, mean ± SD	69.11 ± 16.51	66.11 ± 15.34	69.33 ± 14.24	0.703	0.420	68.66 ± 14.38	64.16 ± 17.07	68.92 ± 14.70	0.213	0.230
IVCd maximum, mean ± SD	1.49 ± 0.49	1.45 ± 0.52	1.71 ± 0.57	0.740	0.005	1.58 ± 0.71	1.57 ± 0.60	1.61 ± 0.46	0.636	0.663
IVCd minimum, mean ± SD	0.44 ± 0.27	0.51 ± 0.36	0.53 ± 0.31	0.665	0.515	0.50 ± 0.34	0.55 ± 0.31	0.51 ± 0.30	0.713	0.709

*E/A* the E(early) to A(late) ratio ventricular velocities; *a´* late (atrial) diastolic mitral annular velocity, *e´* early diastolic mitral annular velocity, *E/e´* ratio of peak early mitral inflow velocity to early diastolic mitral annular velocity, *LVEF* left ventricular ejection fraction; *LVOT* left ventricular outflow tract, *MV* mitral valve, *IVC* inferior vena cava, *IVCd* inferior vena cava diameter

Values are presented as means ± standard deviations. *p*-values correspond to paired *t* tests

## Discussion

Our study found evidence of myocardial involvement in 22.2% of patients who were hospitalized, among whom clinical manifestations ranged in severity from mild elevation of cardiac biomarker levels to myocarditis. Fortunately, these cardiac abnormalities were transient and did not necessitate specific treatment. Impairment of systolic function, characterized by a left ventricular ejection fraction of less than 45%, was found in other studies of severe dengue [3, 6, 18]. In another descriptive and prospective study of 102 paediatric patients with DHF, 10 patients had fulminant myocarditis that necessitated early inotropic drug support for acute heart failure [19]. Prospective studies have reported various incidences of abnormal cardiac involvement in dengue: myocarditis in 15% to 27% of cases [4, 5, 8, 9, 12, 20] and functional cardiac abnormalities in up to 40% [3, 17, 21].

In contrast to these findings, myocarditis was suspected in two of our patients on the basis of clinical information, elevated cardiac enzyme levels, and minimal depression of left ventricular ejection fraction. The difference in the incidence of myocarditis in other reports may be related to dengue severity, which can vary year by year; this study was conducted during a year when severe dengue was not prevalent. Our results are compatible with those of a report of dengue cases in Southeast Asia [22–24] and a report from Sri Lanka [5]. Wichmann et al. [12] showed that 25% of patients with dengue had elevated levels of one or more cardiac biomarkers, such as myoglobin, CK-MB, troponin-T, N-terminal pro B-type natriuretic peptide, and heart-type fatty acid-binding protein. Myocardial involvement may result from a direct effect of the dengue virus or from cytokine-induced immune damage, in which high circulatory levels of pro-inflammatory cytokines cause depression of myocardial function [25, 26]. Another potential mechanism is regional vulnerability to coronary hypoperfusion [27]. Patients with elevated levels of cardiac biomarkers showed more inflammatory activity, such as higher white blood cell counts, but these findings were not statistically significant compared with previous studies [28].

Cardiac involvement in dengue, although often mild, can be severe, progressing to heart failure, according to several reports [3, 11, 29]. Abnormal ECG findings in dengue have been used by some authors to detect cardiac involvement in dengue [5]. We found that no relationship was demonstrated between DF and DHF. Cardiac biomarkers (troponin-T and/or CK-MB) can indicate the presence of cardiac involvement in dengue, especially on the day of defervescence; troponin-T correlated with depressed left ventricular function [6]. Cardiac and haemodynamic parameters are affected by cardiac function, volume status and autonomic responses [27]. Functional cardiac involvement in dengue was found to involve both diastolic and systolic function and was related to severity of plasma leakage [27]. Echocardiographic findings of myocardial injury in DVI have been demonstrated [6, 9, 28, 30, 31]. We found that abnormalities in cardiac parameters were related to the severity of DVI. Decreases in left ventricular ejection fraction, cardiac index and left ventricular diastolic inflow, and elevations in systemic vascular resistance in DHF are likely to be affected by reduced intravascular volume [27]. We found decreases in mitral valve early wave peak velocities, early diastolic mitral annular velocity (e´), left ventricular outflow tract mean and cardiac index in DHF. Lower e´ in this study may reflect diastolic dysfunction, as in previous studies [27, 32]. Cardiac functional assessment with the use of tissue Doppler imaging parameters revealed that e´ was significantly decreased in patients with severe dengue, which may reflect impaired left ventricular relaxation and diastolic defects [27, 33].

Fatal dengue-related myocarditis has also been reported [33]. In a study of adult and paediatric cases in Brazil, the incidence of myocarditis amongst patients with clinical manifestations or elevated biomarker levels was approximately 15% [35]. In a subset of these cases, echocardiographic or magnetic resonance imaging (MRI) findings were abnormal [8, 12]. When dengue-related myocarditis occurs, good supportive care with optimal intravascular volume and fluid maintenance is crucial. According to many studies, myocarditis is transient and self-limiting. In particular, dengue with suspected cardiac involvement should not be treated with iatrogenic fluid overload [18, 35]. The decrease in heart rate on the day of defervescence that has been observed in DVI is attributed to increased parasympathetic activity [36]. In this study, we found that patients with severe DVI are likely to be at risk of cardiac involvement; however, the numbers of patients categorized by severity were small. Further studies are needed to evaluate the risk factors. We recommend echocardiography as the investigation of choice for evaluating haemodynamic status in patients with DVI. We found that DHF was one of the associated risk factors for the development of cardiac involvement in DVI. This finding will increase physicians’ awareness of the possibility of cardiac involvement in patients with DHF.

This study had some limitations. First, we studied a population at only a single center in Thailand; our data may not be representative of all patients with dengue. Second, we studied hospitalized adults with DVI during a period when few cases of myocarditis related to dengue were reported; hence, the results may not be extended to all patients with DVI.

## Conclusions

In this study, the prevalence of cardiac involvement in adults with DVI was 22.2%; manifestations included elevated levels of cardiac biomarkers, transient left ventricular systolic and diastolic dysfunction, myocarditis and pericardial effusion. The functional abnormality was transient and resolved spontaneously by day of follow-up without specific treatment. Myocarditis in patients with DVI was uncommon. We found that DHF is one of the risk factors for the development of cardiac involvement in DVI. Echocardiography is the investigation of choice for evaluating haemodynamic status in patients with DVI, especially in severe cases.

## Supplementary Information

Below is the link to the electronic supplementary material.

**Additional file 1:Table S1.** Current medication in dengue patients.

## Data Availability

All included data are available from the corresponding author upon request.

## Abbreviations

a´: Late (atrial) diastolic mitral annular velocity
ALT: Alanine aminotransferase
AST: Aspartate aminotransferase
BMI: Body mass index
CI: Confidence interval
CK-MB: Creatine kinase–myocardial band
DF: Dengue fever
DHF: Dengue haemorrhagic fever
DSS: Dengue shock syndrome
DVI: Dengue virus infection
e´: Early diastolic mitral annular velocity
E/A: The E(early) to A(late) ratio ventricular velocities
ECG: Electrocardiography
E/e´: Ratio of peak early mitral inflow velocity to early diastolic mitral annular velocity
ELISA: Enzyme-linked immunosorbent assay
ESC: European society of cardiology
IgM: Immunoglobulin M
IgG: Immunoglobulin G
IQR: Interquartile range
IVC: Inferior vena cava
IVCc: Inferior vena cava collapsibility index
IVCd: Inferior vena cava diameter
IVCdmax: The maximum inferior vena cava diameter
IVCdmin: The minimum inferior vena cava diameter
LVEF: Left ventricular ejection fraction
LVOT: Left ventricular outflow tract
MRI: Magnetic resonance imaging
MV: Mitral valve
MV-A: MV flow A-wave velocity
MV-E: MV flow E-wave velocity
MV-E/A: The E(early) to A(late) ratio ventricular velocities
NS1 antigen: Nonstructural protein1 antigen
OR: Odds ratio
SD: Standard deviation
Trop-T: Troponin-T
WBC: White blood cell counts
WHO: World health organization
