# Supplementary material for: Cardiac evaluation in adults with dengue virus infection by serial echocardiography
Source: BMC Infect Dis. 2021 Sep 10;21:940. doi: 10.1186/s12879-021-06639-x (PMC8431916; doi:10.1186/s12879-021-06639-x)
Supplement: Supplementary file 1 — Additional file 1:Table S1. Current medication in dengue patients. [file 12879_2021_6639_MOESM1_ESM.docx]

**Additional file 1: Table S1. Current medication in dengue patients**

| **Characteristic** | **Total (*N* = 81)** | **DF (*n* = 39)** | **DHF (*n* = 42)** |
| --- | --- | --- | --- |
| Angiotensin-Coverting Enzyme  inhibitors; ACEI (n) | 2 | 2 | 0 |
| Calcium channel blocker (n) | 2 | 2 | 0 |
| Thaizide diuretic (n) | 1 | 1 | 0 |
| Beta blocker (n)  Biguanides (n)  Statin (n)  Aspirin (n)  Thiazolidinediones (n)  Alpha-glucosidase inhibitor (n)  Liraglutide (n) | 1  2  3  1  1  1  1 | 1  2  2  1  1  1  1 | 0  0  1  0  0  0  0 |
